# Fiber Bragg Grating Sensors for the Oil Industry

**DOI:** 10.3390/s17030429

**Published:** 2017-02-23

**Authors:** Xueguang Qiao, Zhihua Shao, Weijia Bao, Qiangzhou Rong

**Affiliations:** School of Physics, Northwest University, No.229, Taibai Road, Xi’an 710069, China; 2013optics_szh@stumail.nwu.edu.cn (Z.S.); vj199107@163.com (W.B.)

**Keywords:** oil and gas industry, fiber Bragg grating, seismic exploration, high temperature, high pressure, acoustic wave, well-logging field

## Abstract

With the oil and gas industry growing rapidly, increasing the yield and profit require advances in technology for cost-effective production in key areas of reservoir exploration and in oil-well production-management. In this paper we review our group’s research into fiber Bragg gratings (FBGs) and their applications in the oil industry, especially in the well-logging field. FBG sensors used for seismic exploration in the oil and gas industry need to be capable of measuring multiple physical parameters such as temperature, pressure, and acoustic waves in a hostile environment. This application requires that the FBG sensors display high sensitivity over the broad vibration frequency range of 5 Hz to 2.5 kHz, which contains the important geological information. We report the incorporation of mechanical transducers in the FBG sensors to enable enhance the sensors’ amplitude and frequency response. Whenever the FBG sensors are working within a well, they must withstand high temperatures and high pressures, up to 175 °C and 40 Mpa or more. We use femtosecond laser side-illumination to ensure that the FBGs themselves have the high temperature resistance up to 1100 °C. Using FBG sensors combined with suitable metal transducers, we have experimentally realized high- temperature and pressure measurements up to 400 °C and 100 Mpa. We introduce a novel technology of ultrasonic imaging of seismic physical models using FBG sensors, which is superior to conventional seismic exploration methods. Compared with piezoelectric transducers, FBG ultrasonic sensors demonstrate superior sensitivity, more compact structure, improved spatial resolution, high stability and immunity to electromagnetic interference (EMI). In the last section, we present a case study of a well-logging field to demonstrate the utility of FBG sensors in the oil and gas industry.

## 1. Introduction

By 2017, according to forecasts, the world’s economies will demand about 33 million barrels per day [1,2,3,4,5,6,7,8,9,10,11,12,13,14]. Cost-effective oil production is becoming more important than ever, not only to the profit-making oil corporations and nations, but also to countries, communities and individuals around the world. Advances in technology are required in key areas of the oil industry, most notably in reservoir exploration and in oil-well production management, to increase yield and profit. The application of new technology in oil refinery processes and in monitoring and preventing leaks in oil and gas transport pipelines will also have an impact on future global oil production. In the early days of the oil industry, oil explorers would guess the location of subsurface reservoirs by recognizing surface features that marked their presence. Today, however, the majority of these shallow oil traps have been exploited and explorers must now seek oil in deep and offshore reservoirs. Considering the remote locations of such reservoirs and the logistics and expense of offshore drilling, these “missed wells” add significant cost to the development of successfully located reserves. The applications of new technology in the form of seismic surveying, three-dimensional imaging and virtual reality can now allow geologists to accurately pin point where to drill for oil. This significantly reduces the number of exploration wells that need to be drilled and allows for more careful planning and drilling of development wells. The availability of permanent downhole information such as temperature, pressure, acoustic and vibration parameters within wells is one of the key elements to improve reservoir management. The key to improved management is better prediction and control of the distribution and movement of oil, gas and water within the reservoir.

In the 1960s, optical fibers with cylindrical waveguide structures were initially designed solely for the transmission of light. To date, optical fiber technology has had an enormous impact on modern telecommunication systems [15,16,17,18,19,20,21,22]. The intrinsic properties of the light transmitted along an optical fiber, such as its intensity, phase, spectrum, frequency and polarization can be affected by the surrounding environment (e.g., temperature, strain, refractive index (RI), magnetic field and biochemical parameters), making optical fiber a good candidate for sensing applications. Over the last 40 years, fiber sensing as a smart technique has attracted great interest in multiple fields of application due to their advantageous properties of electrical insulation, EMI immunity, intrinsic safety, passive operation (no need for electrical power), possibility of remote and multiplexed operation, small size and light weight, integrated telemetry (the fiber itself is a data link), wide bandwidth and high sensitivity. Fiber sensors can take advantage of diverse smart processing technologies and the novel materials [23,24,25,26,27]. The sensors mentioned above operate via a range of sensing mechanisms including [28,29,30,31,32,33,34,35,36,37,38,39]: interferometry (measuring optical phase), intensity (measuring the change in the guided light power); spectrometry (measuring changes in optical resonant frequency or wavelength of an optical cavity); polarimetry (measuring polarization state of the guided light); and diffraction (measuring frequency of lightwave interfering with a periodic structure). Among optical fiber sensing methods, fiber Bragg grating (FBG) sensors, in particular, have attracted great interest because of their unique properties, which open up many opportunities for single-point sensing of many parameters in hard-to-reach spaces, with controllable cross-sensitivities and very compact size, making them suitable for embedded measurement. These outstanding properties make FBGs especially well adapted for well-logging and seismic exploration in oil and gas fields. There is presently much scope for growth and further commercialization of FBG sensors in the oil and gas industry.

The photosensitivity of optical fiber was discovered by Ken Hill et al. at the Canadian Communications Research Center in 1978 [40]. Hill’s group fabricated the first FBGs in germania-doped silica fiber using visible argon ion laser radiation. FBGs are created by a periodic perturbation of the RI along the fiber length, formed by exposure of the core to an intense optical interference pattern. The inscription of the interference pattern can be achieved using a range of light sources including KrF* excimer lasers at 248 nm, excimer ArF* lasers at 193 nm and continuous UV sources at 240 nm [41,42,43,44,45,46,47,48]. The refractive index change in the fibers is dependent on the absorption band of defects in doped-silicate fiber [49,50]. Refractive index modulation depth can also be increased by hydrogenating the fiber prior to inscription. If the power of the laser illumination is increased from the megawatt to the gigawatt range, the probability of the occurrence of two-quantum photo processes in the irradiated medium greatly increases. Two-photon processes include two-step absorption (two-quantum absorption through an intermediate electronic state) and two-photon absorption (two-quantum excitation through a virtual-state). Two-photon effects can increase the modulation depth of the FBG thereby creating a stronger FBG. For example, to create strong gratings, one can use a femtosecond laser such as a Ti: sapphire laser at 800 nm for FBG inscription. Such two-photon inscription can create strong gratings without the need for hydrogenation of the fiber [51,52,53,54,55,56,57,58,59].

The laser radiation can induce two regimes of RI according to the laser intensity. Irradiation of the fiber at relatively low optical intensity creates a RI change in the fiber’s core that can be partially or completely reversed at relatively low temperatures. That index change can be annealed out at temperatures below 700 °C (so-called type I gratings). At relatively high illumination intensity, above the self-focusing threshold, a permanent RI change is induced by multiphoton and avalanche ionization causing plasma formation. These so-called type II gratings can withstand temperatures higher than 700 °C without degradation [60]. There are two principal approaches for inscription of FBGs using femtoscond lasers: directly writing and side-illumination [61,62,63,64]. In the direct writing method, a tightly focused femtosecond laser beam is precisely stepped axially along the fiber, with precise control of the step size, the laser’s power and the exposure time at each step. The interferometric technique using a phase mask requires careful control of the distance between the phase mask and fiber as well as exposure time and power.

The traditional FBGs have been employed as sensors for multiple parameters, such as temperature, pressure, acoustic wave amplitude, frequency and phase, and static and dynamic strains, by the recovery of wavelength information [65,66,67,68]. Changes in transmitted and reflected optical spectra can be used to interrogate physical and chemical parameters [69,70,71,72]. In order to improve the sensitivity of intensity-referenced sensors, diverse post-processing operations are utilized to couple light from the core mode into cladding modes [73,74,75,76]. These cladding modes have unique mode field shapes and distribution over the cross-section of fiber, and thus they can respond differently to perturbations within and outside the fiber. The FBGs mentioned above present a common property: they are all written in cylindrically symmetrical fibers. However, when a FBG is written in a polarization-maintaining (PM) fiber, two orthogonally polarized modes will propagate in the fiber core, and two different resonant wavelengths are seen in the reflection spectrum [77,78,79,80]. The two polarized modes can respond differently to fiber perturbations such as transverse strain [81], twist or torsion [82] and temperature [83]. The PM-FBG sensor has clear orientation-dependence and thus can be used as a two-dimensional sensor. The writing of these FBG inscriptions has traditionally been achieved using ultraviolet exposure. Alternatively, 800 nm femtosecond laser inscription has become a popular technique. Bragg grating structures can also be inscribed in the cladding of a fiber and in coreless optical fibers [25]. Among the novel grating structures the cladding FBGs have demonstrated outstanding performance as power-referenced fiber bending sensors, which make use of highly reflective cladding mode resonances, and narrow resonance bandwidths. Such novel FBGs further enrich the short grating family and also provide insight into mode recoupling in a mode-mismatched structure. Furthermore, because of the high peak power of femtosecond lasers, type II FBGs have the ability to withstand temperatures as high as 1100 °C, making them suitable for ultra-high temperature measurements.

FBG sensors have now become mainstream sensors in oil and gas exploration, leading to increased demand for better fiber-based sensors. This paper reviews FBG-based sensors for well-logging, and, in particular, introduces our group’s work in this area. The research work described here has been carried out in our institutes, the Photoelectric Technology and Application International Research Center of China and the Key Laboratory Cultivation Base Construction of Photoelectric Technology and Function Materials of Shaanxi Province. For more than twenty year sour group has been working on fiber-optic sensors, especially FBG sensor technology, their applications in the oil and gas industry. Our group’s fiber sensing instruments for measuring pressure and strain have been tested successfully in oil and gas fields, such as the Qingyang-Xianyang pipeline in 2006, the Liaohe oil field in 2011, the Changqing oil field in 2012 and by the China Petroleum Well-logging Company in 2015.

## 2. Functional FBG Sensors

### 2.1. Vibration and Acceleration Sensors

**Figure 1:**
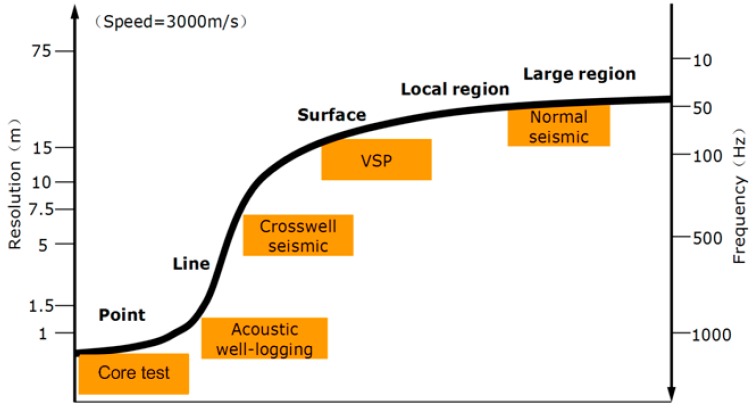
Frequency band range and longitudinal resolution of multi scale geophysical data [100,101,102].

The detection and measurement of vibration is crucial for oil and gas seismic exploration. Recently, fiber-optic vibration (or, equivalently, acceleration) sensors have received more attention than their electrical counterparts because of their inherent superiority, as described above [84,85,86,87,88,89,90,91,92,93,94,95,96]. In particular, FBG accelerometers have undergone rapid development and have established themselves as the leading technology, compared to other fiber sensor technologies. In order to increase vibration sensitivity and response frequency of FBGs, the gratings are frequently coupled to mechanical components such as special elastic material layers, plate-beams and flat circular diaphragms. In these sensors, the detection of vibration information is achieved through the wavelength interrogation of FBGs. Of course, the intensity of reflection spectrum can also be used to monitor vibration by employing side-band filtering techniques [97,98,99]. These sensors provide high dynamic strain sensitivity and realize the compensation of temperature over a full range. However, problems such as the power disturbance of light in the sensing system, and multiplexing capability, will require further work. For oil well exploration, the seismic waves cover a frequency bandwidth from several dozens to hundreds of Hz [100,101,102], as shown in Figure 1.

**Figure 2:**
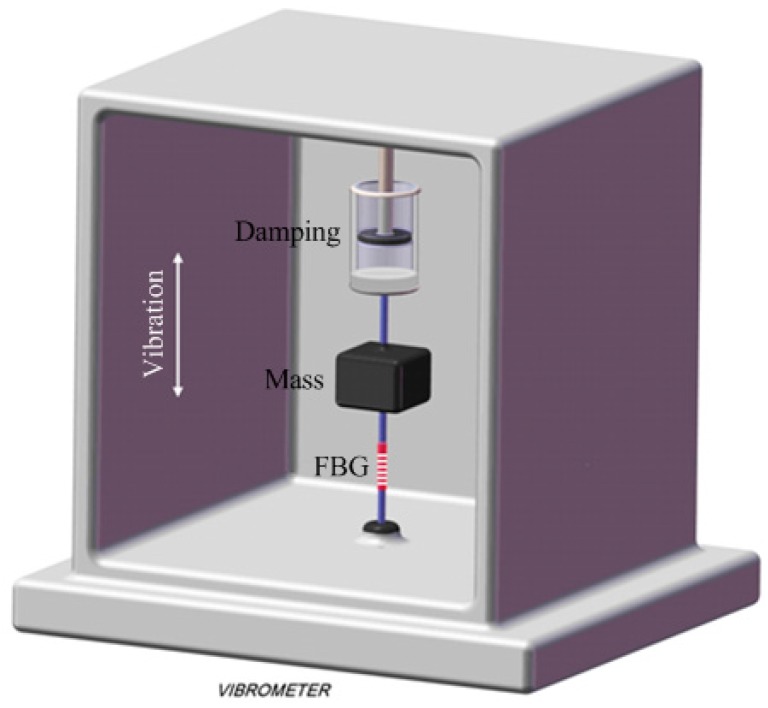
Mechanical model of a FBG accelerometer.

The naked FBGs themselves are usually insensitive to vibration and manifest a single resonant frequency. Thus they need to be made more sensitive with the help of mechanical transducers of different structures, which improve the FBGs’ amplitude-frequency performance of the response to vibration. In the testing process, the parameter of acceleration is generally employed to characterize the vibration of the objects. In Fiber Bragg grating vibrometers, the FBGs themselves play the role of an elastic element. The mass displacement induced by the vibration is transferred to the sensing element, which then strains the FBG, resulting in the spectral shift of FBG’s resonant peak. Measurement of the amplitude and frequency of vibration can be achieved by the recovery of FBG wavelength information. The mechanical model of a simple FBG accelerometer can be modeled as a mass-spring system with damping, as show in Figure 2, where *m* is the mass of mass bulk, *k* the stiffness coefficient of the FBG, *c* the damping coefficient, a the acceleration in the vertical direction (In the following mathematic derivation the vibration system is simplified into a mass-spring (mass + FBG) system and the damping coefficient is not considered).

When vibration occurs, the mass, *m*, senses an inertial force with the opposite direction to that of initial vibration. The FBG is strained alternatingly in extension and compression, with time-varying strain ε. The relationship between the axial strain and inertial force is expressed by:

(1) $$ma=EA\varepsilon =EA\frac{\Delta L}{L},$$

where *E* is the Young’s modulus of the fiber, *A* is the cross-sectional area of fiber, *L* is the length of the FBG and Δ*L* is the length change of FBG. The relation between the wavelength variations Δ*λ_B_* versus strain applications is written as:

(2) $$\Delta \lambda_{B}=\left(1-P_{e}\right)\varepsilon \lambda_{B},$$

where *P_e_* is the Poisson’s coefficient, and *λ_B_* is the central FBG wavelength.

According to the Equations (1) and (2), the following equation is derived:

(3) $$\Delta \lambda_{B}=\left(1-P_{e}\right)\frac{ma}{EA}\lambda_{B}.$$

The acceleration sensitivity of FBG can be written as:

(4) $$S=\frac{\Delta \lambda_{B}}{a}=\left(1-P_{e}\right)\frac{m\lambda_{B}}{EA}.$$

According to the Hooke’s law, the stiffness coefficient of the FBG is expressed as:

(5) $$k=\frac{ma}{\Delta L}=\frac{EA}{L}.$$

From elementary mechanics, the resonant frequency *f* of a mass-spring system (in which the FBG acts as a spring) is given by:

(6) $$f=\frac{1}{2\pi }\sqrt{\frac{k}{m}}=\frac{1}{2\pi }\sqrt{\frac{EA}{mL}},$$

The above analysis shows that the acceleration sensitivity of FBG is inversely proportional to the square of the resonance frequency of (FBG + mass) system. In the above model, the FBG is the elastic element that experiences the strain force directly, acting as a mechanical spring. This configuration suffers from poor dynamic range and mechanical reliability since the FBG can only tolerate a maximum strain of about 1% (10^4^ με). For improved mechanical robustness, it is necessary to couple the FBG to a mechanical transducer. The mass moves relative to the base, in response to vibration and transmits its motion to a mechanical transducer such a cantilevered beam or diaphragm [25]. This mechanical movement couples to the FBG, whose Bragg wavelength changes accordingly. The elastic element in the accelerometer is mechanical device instead of FBG, and acts as a bridge that transmits the vibration to FBG. The mechanical structure can be freely designed to achieve the desired sensitivity and resonant frequency.

#### 2.1.1. One-Dimensional Vibration Sensors

**Figure 3:**
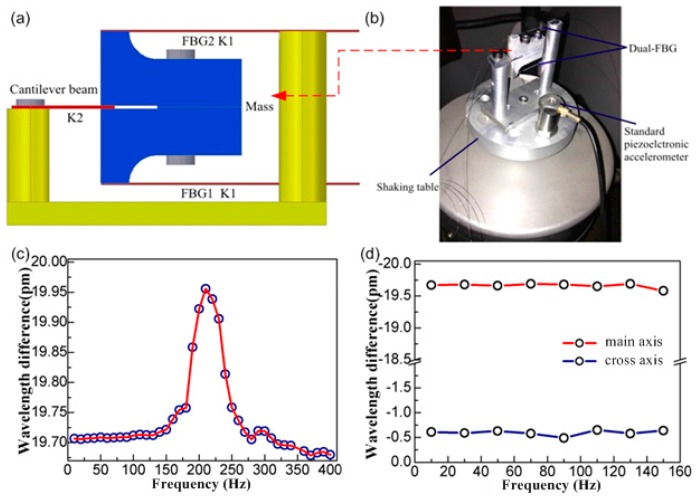
(**a**) Scheme structure of the proposedaccelerometer; (**b**) Photograph of the accelerometer and testing exciter; (**c**) Amplitude-frequency response of accelerometer; (**d**) Transverse direction dependence of resonance wavelength difference of FBGsunder the acceleration excitation of 1.5 G.

The 1D accelerometer is only required to respond to vibration along a single axis. The key to success is to transfer vibratory motion along the desired axis only, to the FBG by appropriate design of the mechanical transducer. At the same time, the mechanical transducer also determines the sensitivity, the mechanical response frequency and bandwidth of the sensor. In previous work, our group proposed a hybrid cantilever beam-based FBG accelerometer [103], as shown in Figure 3. The moving component of the sensor consists of an elastic rectangular cantilever beam and a pair of relatively massive L-shaped aluminum blocks. The cantilever beam, made from spring steel, supports the mass block formed by bolting the aluminum blocks back-to-back. FBG1 and FBG2 are pre-stressed each with one end fixed to the upper and lower surfaces of the mass block, respectively, and to a support pillar at their other ends. When vibration is applied vertically, the mass moves up and down, compressing and stretching FBG1 and FBG2 in opposite senses. In this work, the cross-axis sensitivity is decreased to 3.2% thanks to the dual-beam’s directional vibration responses, as shown in Figure 3d. Furthermore, the temperature is self-compensated based on the same responses to temperature of two reflection wavelengths. Finally, a considerable sensitivity of 215.6 pm/G is achieved stably over the frequency range of 10 Hz to 150 Hz.

**Figure 4:**
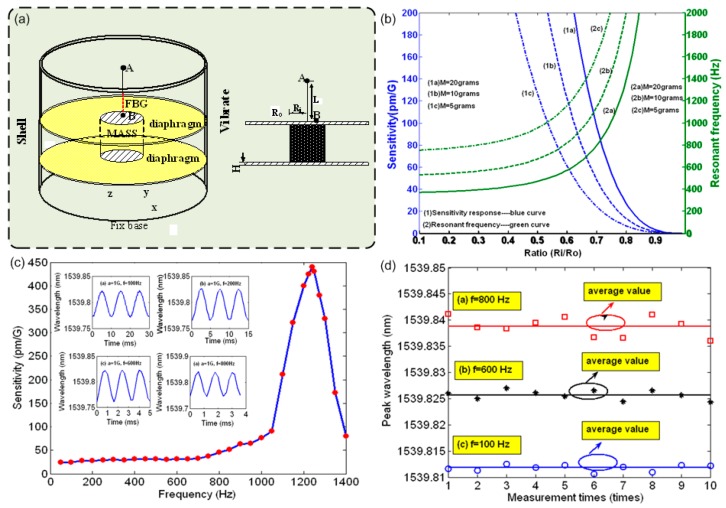
(**a**) Schematic diagram of FBG accelerometer based on double diaphragms; (**b**) Simulated sensitivity and resonant frequency as a function of mass diameter; (**c**) Frequency response of accelerometer (insets show the time-domain variation of resonant wavelength for vibration frequencies 100, 200, 600 and 800 Hz); (**d**) Stabilities of the accelerometer under the vibrations with the different frequencies.

In the above work, the resonant frequency of the accelerometer is dependent on the mass of the mass block and the characteristics of the cantilever beam. The measurement of vibration at higher frequency requires different sensor structures. For example, another accelerometer for higher frequency vibration we demonstrated employed a cooper diaphragm-shaped structure [104]. As shown in Figure 4a, a cylindrical mass is sandwiched between two horizontal diaphragms mounted inside a vertical cylindrical shell. A FBG is pre-tensioned and attached to a fixed upper cover plate and the mass block, at points A and B respectively. The mass moves with the environmental vibration along the vertical (z) direction and repeatedly stretches and compresses the FBG. Factors that influence the sensitivity and frequency response of the accelerometer are the inertial mass (*M*), the ratio of the mass radius to diaphragm radius (*R_i_/R_0_*) and Young’s modulus of the diaphragms. The demonstrated mechanical resonant frequency of the accelerometer was above 1200 Hz thanks to the dual-diaphragm-shaped structure and the large Young’s modulus of the diaphragm. In addition, the inertial mass is fixed between the two diaphragms. The vibration response occurs only along the z-direction, i.e., the motion of the mass has only a single degree of freedom. The directivity of the sensor effectively avoids cross-talk from vibration in the x- and y-directions. Varying the mass changes the amplitude-frequency response of the sensor, as shown in Figure 4b. Figure 4c shows the sensitivity and resonant frequencies at masses of 5, 10 and 20 g. The sensor has demonstrated good stability over the whole frequency bandwidth, here taking several cases at the vibration frequency of 100 Hz, 600 Hz and 800 Hz, as shown in Figure 4d.

In the section above, we considered two examples of 1D FBG vibration sensors. Many other 1D vibration sensors for applications in oil and gas exploration, bridges and building have been reported. For example, Zhang et al. reported an extensional FBG-based accelerometer with a resonant frequency of 16.7 Hz and sensitivity of 410.7 pm/G. The device realized acceleration measurement ranging from 0 to 5 G [105].

Mita et al. reported a cantilever beam-based FBG accelerometer with a sensitivity of 498 με/G and resonant frequency of 49.2 Hz [106]. Weng et al. reported a FBG accelerometer formed by two L-shaped rigid cantilever beams and diaphragm, in which a FBG hangs between the beams to avoid chirping the FBG [107]. The accelerometer had a sensitivity of 100 pm/G, a frequency response range of 10 to 120 Hz, and cross-axis sensitivity of 5%. Todd et al. reported an accelerometer with a small cross-axis sensitivity of 1% by using beam-plates. The structure supports the sensor has a high resonant frequency of 300 Hz [108]. An FBG-based accelerometer consisting of a mass resting on a layer of compliant material supported by a rigid base plate has shown a high resonant frequency of 2 kHz [109]. These accelerometers all have good potential as one-dimensional vibrometers, but are unable to detect two-dimensional (2D) vibration.

#### 2.1.2. Two-Dimensional Vector Vibration Sensors

Orientation information is one of the critical issues for vibration (acceleration) measurement in various engineering applications, especially when the vibration source is unknown. One idea for 2D vector vibration detection is to employ two orthogonal one-dimensional vibration sensors. After combining the orthogonal vibration information collected by the two sensors, the vibration direction in a plane can be determined. However, the need for multiple sensors increases complexity and bulk. Furthermore, the precision of direction determination depends on the maintaining precise orthogonality between the axes of the individual sensors over a long time, often in challenging environments. A preferred solution is to employ FBG sensors with inherent 2-dimensional measurement capability. Since cladding modes have unique mode field shapes and polarization-dependent transmission spectra, they react differently to perturbations inside and outside the fiber (especially to transversal strain), providing good potential for high-sensitivity vibration amplitude, frequency and direction measurement. Fiber grating vibroscopes, using tilted fiber Bragg gratings (TFBGs) have been reported [110,111]. The grating tilt breaks the cylindrical symmetry of the fiber and launches linearly polarized light into the fiber cladding at orthogonal orientations relative to the tilt plane, allowing for vectorial vibration information to be retrieved. Alternatively, gratings written in polarization-maintaining fiber (PMF) [112,113] have been shown to function as 2D vibration sensors. The cladding modes are divided into two polarization states which show orthogonal responses to vibrations aligned with the fiber’s X- and Y- polarization directions. Sensors based on TFBGs and PMF-FBG are both smart 2D vector vibration sensors. Although the two cases above are capable of the 2D vibration detection, the small signal-to-noise ratio of cladding modes (owing to the large coupling loss and transmission loss) decreases the precision of the sensor, and the broad occupancy rate in the spectrum makes multiplexing of multiple sensors problematic.

**Figure 5:**
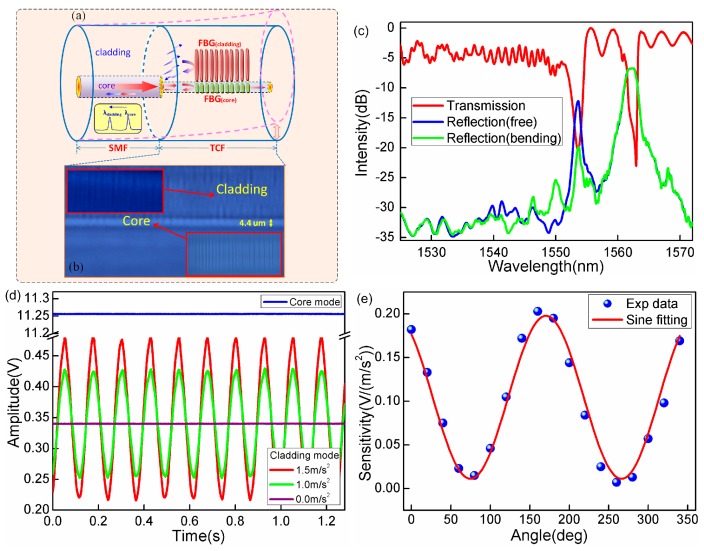
(**a**) Schematic diagram of TCF-FBG directional accelerometer; (**b**) Image of TCF-FBG, insets show the zoomed images of gratings in fiber cladding (up) and fiber core (bottom); (**c**) The transmission spectrum of TCF-FBG with two well-defined resonances; (**d**) Real-time power output of cladding mode and core mode (blue curve) under the same vibration condition; (**e**) Angular dependence of acceleration responsivity of sensor.

In order to avoid the uncertainties associated with the reproducibility and spectral quality of tilted gratings, our group has developed a new vectorial vibration sensing mechanism. The sensor is created by writing a conventional FBG in the cladding of a short piece of thin-core fiber (TCF) spliced to standard single-mode fiber (SMF), as shown in Figure 5. The key to the success of this device lies in the inherent mismatch between the cores of the two fibers (which allows the coupling of core modes into cladding modes) and the precisely localized grating inscription within the fiber cladding (thanks to highly focused femtosecond laser inscription and photo-sensitivity of the TCF cladding). We show below that this device has superior spectral qualities that facilitate sensor interrogation and are significantly simple to fabricate. The grating inscription region is close to the core–cladding interface of the TCF, with the most of the grating within the fiber cladding and partially extending into the fiber core. With this configuration, two well-defined resonances in reflection have been achieved, which originate from the gratings in the core and the cladding, respectively, as shown by the “FBG core” and “FBG cladding” marked in Figure 5a. 

The cladding Bragg resonance which is located about 8.8 nm away (on the short wavelength side) demonstrates high polarization dependence and reacts strongly to small fiber bending. This cladding resonance corresponds to the mode coupling from the core of the input SMF to the cladding of the TCF with low loss at the splice point. Then the cladding mode is reflected by the cladding FBG and partially is recoupled back to the input SMF, and eventually returns to the interrogation system. Most importantly, in contrast to earlier reports of ghost modes in tilted FBGs (a group of low-order cladding modes with overlapping spectra and complex polarizations [114,115]), the recoupled cladding mode in TCF-FBG shows clear polarization dependence because of the asymmetrical distribution of the cladding FBG along the fiber cross section. As a result, we achieved strong orientation dependence of vibration measurement in a low cost sensor. A further advantage is that unwanted power fluctuations and temperature perturbations can be referenced out by monitoring the fundamental mode resonances, which are unaffected by bending.

The reflection intensity of the thin-core fiber cladding FBG sensor described above is superior to that of the TFBG and PMF-FBG vibration sensors, but it is still small and is also easily affected by the surrounding RI since the grating is distributed over the fiber cladding [25,116]. To overcome this shortcoming, we fabricated a special fiber with four-cladding layers surrounding the core. A cladding FBG is written into the innermost photosensitive cladding of the fiber. The multi-layer cladding structure strongly couples core-guided light into cladding modes, which are able to propagate in the cladding with low loss because of the weak waveguiding in the inner cladding. The cladding mode resonance of the cladding FBG is stronger than that of core FBG. The index profile of the cladding layers is optimized to increase the loss of inner cladding-guided light to the outer cladding layers as the fiber is bent. This property increases the cladding FBG’s response to fiber bending relative to that of the cladding FBG in single-clad fiber. The result is a more bend-sensitive directional accelerometer.

**Figure 6:**
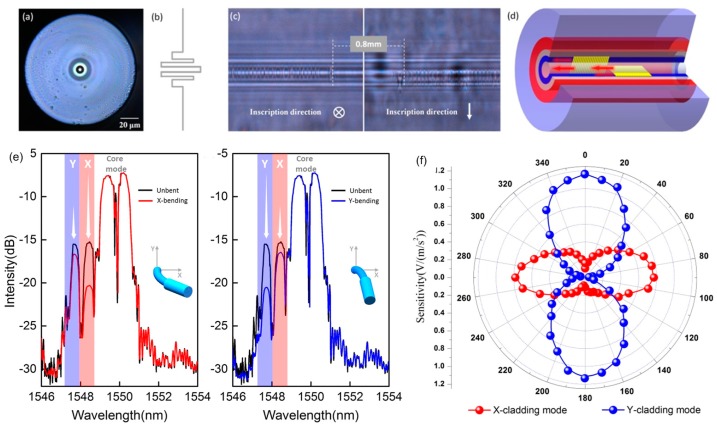
(**a**) Cross-sectional photomicrograph of the MCF-FBG; (**b**) Refractive index profile of the MCF-cross section; (**c**) Longitudinal photomicrograph of gratings inside MFC; (**d**) Schematic of the vector grating; (**e**) Spectra of the vector-FBG; (**f**) Angular dependence of acceleration responsivity of this sensor.

We recently demonstrated a 2D vector vibrometer based on direct power detection of orthogonal cladding FBG reflections. The sensing probe consists of a compact structure in which a short section of the multi-clad fiber (MCF) containing two orthogonally cascaded cladding FBGs is spliced to SMF with self-alignment process. We wrote the two gratings, azimuthally separated by 90°, using femtosecond laser side-illumination, taking care to ensure the two grating inscriptions are precisely positioned and compact in size, as show in Figure 6c,d. With such configuration, as shown in Figure 6e, the reflection spectra show pairs of well defined cladding resonances from the internal cladding (two weak resonances at shorter wavelength side) and from the fiber core (two strong resonances at longer wavelength side). Most importantly, the cladding resonances are strongly directional sensitive to the small fiber bending, and the orientation of vibration can be determined in a two dimensional plane. Any optical power fluctuations are referenced out by core Bragg resonances that are insensitive to fiber bending. When the dual cladding FBGs work as an accelerometer, the intensities of the cladding mode resonances present strongly orthogonal responses thanks to the two orthogonally positioned gratings in the cladding. Figure 6f demonstrates the angular dependence of acceleration responsivity of sensor. In future work, the 2D direction information can be achieved by analyzing this 2D vibration response.

Table 1 summarizes the key parameters of several types of reported FBG vibration sensors. For field applications in the oil and gas industry, packaging of the sensor is important, and appropriately designed package can enhance the sensitivity of the vibrometer. In addition, the further try of using the FBG for three-dimensional vibration measurements will be expected by recovering the wavelength information of orthogonal cladding FBGs besides the intensities interrogation of them.

### 2.2. High Temperature Sensors

Temperature information is a key parameter in oil and gas well-logging and health monitoring of pipeline fields, especially for deep and ultra-deep well-logging. It is necessary to permanently monitor high temperature in deep wells. FBG temperature monitors need heat-resistant packaging materials to maintain calibration stability in the high temperature environment over long periods. To date, many techniques have been developed to improve the high temperature resistance of FBGs, such as tailoring the glass composition [122,123], specific thermal cycling or annealing processes of the gratings [124,125], and inscribing gratings using femtosecond lasers [59,126,127]. The measurements of high temperature up to 1100 °C have been realized, but the researchers still seek new methods to simplify the fabrication process and reduce costs.

**Figure 7:**
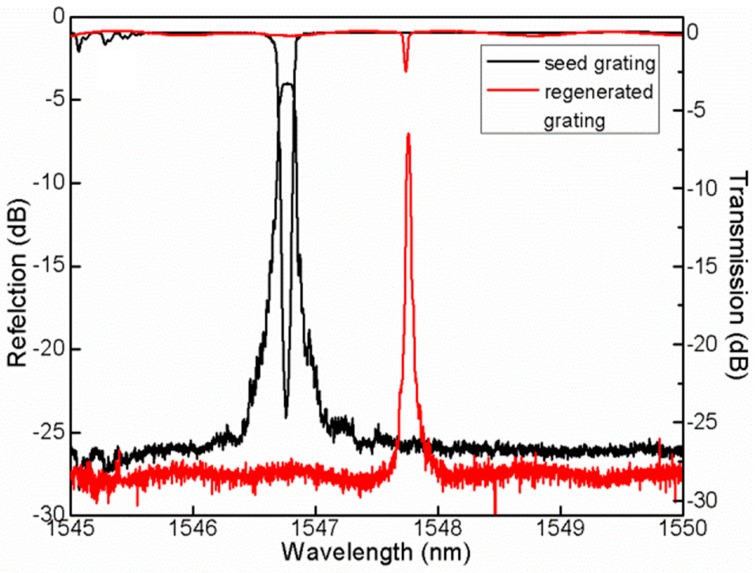
Reflection and transmission spectra of seed gratings (black curve) and regenerated fiber Bragg gratings (red curve).

Regenerated fiber Bragg gratings (R-FBGs), which take advantage of a simple thermal annealing process, have successfully achieved operating temperatures up to 1100 °C. During the thermal regeneration process, a seed grating is gradually erased as the annealing temperature rises towards the “regeneration point”. Continued annealing at increased temperature causes the grating to grow again [128,129,130]. Regeneration of the grating is aided by use of high germania-content fiber and hydrogen loading prior to inscription of the seed grating [131], or by using helium-loaded germanosilicate optical fiber [132]. After regeneration, the RFBG shows narrower spectral bandwidth than the seed FBG, which is helpful in improving the filtering technology and sensor multiplexing, as shown in Figure 7. However, the reflectivity of the RFBG is always lower than that of its seed FBG, although many methods have been tried to improve the reflectivity. Another problem is that the heating process is complex, and it is necessary to monitor and control the spectral response during the regeneration process. RFBGs are mechanically weaker than conventional FBGs following the regeneration, which requires high temperatures, up to 800 °C. The mechanical degradation limits application of RGBGs in well-logging.

**Figure 8:**
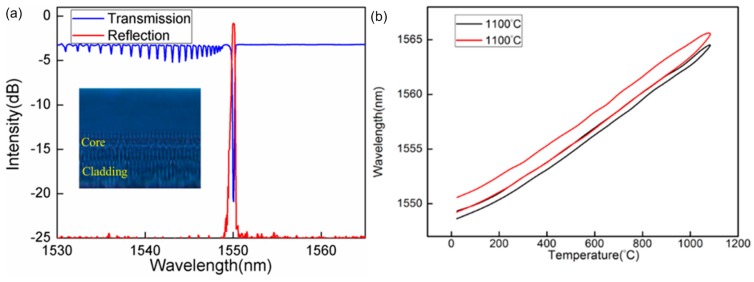
(**a**) Reflection and transmission spectrum of FBG written in a SMF (inset is imaging photo of FBG); (**b**) FBG wavelength versus temperature up to 1100 °C.

In the last few years, FBG fabrication using femtosecond laser has attracted considerable attention from researchers. Smelser et al. realized the formation of two grating types in SMF-28 fiber by focusing 125 fs, 0.5–2 mJ pulse laser through a phase mask onto a fiber sample [133]. The first type, designated “type I-IR”, appears below the damage threshold of the fiber material. The further thermal testing suggests that this I-IR grating is formed by a nonlinear absorption process, possibly resulting in the formation of color centers in the germanosilicate core glass. The second type, specified as “type II-IR”, appears with lased white light formation on the fiber. The type II-IR grating is most likely a consequence of damage to the germanosilicate core glass following ionization, and is possible for these gratings to withstand high temperatures. Thermal stability tests show that femtosecond laser-inscribed type II-IR FBGs exhibit excellent stability at temperatures slightly in excess of 1000 °C. This thermal stability has been attributed to the modification of the fiber core glass by the high peak power and the effects of nonlinear light-material interaction under irradiation by the femtosecond laser. On the support of the phase mask technique, the FBGs inscription over many kinds of fiber are realized easily. Compared to the conventional FBGs written using UV lasers, larger refractive index modification can be induced in fibers by femtosecond laser for its ultrashort pulse width and high peak power. Both the reflectance and transmission spectra of conventional FBGs are complementary and do not present obviously coupling loss. As for the high-temperature stability, conventional FBGs possess poor stability of temperature and they can be wiped out at high temperature below the melting transition temperature of silica fiber. But type II-IR FBGs by fetomsecond laser perform better and the high-temperature stability can be up to 1000 °C. Besides, fs-written type II-IR FBGs have the shorter lengths and higher reflectivities due to a larger refractive index contrast. On the other hand, the inscription of FBGs by femtosecond laser also brings some drawbacks, such as wide spectral bandwidth, low mechanical strength, large birefringence effect and cladding modes resonances, etc. Figure 8a shows the reflection and transmission spectrum of FBG written in a SMF. Due to the large spectral content of the femtosecond pulse after passing through the phase mask, it would be broadly dispersed and the energy spreads over a large area. As a result, some cladding modes resonances appear in the transmission spectrum, waiting for the further technique to eliminate these unexpected resonances.

Figure 8b shows the wavelength responses of FBGs under several heating and cooling temperature cycles up to 1100 °C (and then down to 25 °C). Although the heating and cooling wavelength response curves are parallel, a clear separation exists between them. This significant unrepeatability (thermal hysteresis effect) occurs with wavelength differences over the heating and cooling processes at temperatures around 1100 °C or higher. High thermal stability of the FBGs (even if type II-IR gratings, which are the most temperature resistant) is difficult to maintain at temperatures near and above 1100 °C or higher [134,135], because the gratings rapidly decay. One of the hurdles to achieving improvement in thermal stability lies in the fact that the residual stress exists in all optical fibers during their formation process, which has negative effects on the fiber reliability and grating quality and consequent thermal stability. When the FBG experiences high temperature, the nonequilibrium glass structure will gradually transition to an equilibrium liquid (a collection of smaller units). At the high temperature, the configuration entropy of the smallest unit of the liquid undergoes a relaxation process, and the activation barrier is overcome by the smallest units in order to relax. The relaxation of internal stresses of silica fiber obeys the equation of De Bast and Gilard:

(7) $$\sigma =\sigma_{0}\times \exp{\left(-at\right)}^{b},$$

where *σ*_0_ is original internal stress of fiber, *b* is constant and *a* depends on temperature [136]. Therefore, with the temperature rising, the relaxation of “frozen” stress (resulting from the density of β-quartz and the O-Si-O changes [137]) is speeded up. At the temperature higher than 1100 °C, the units of liquid can be rearranged to release internal stress of silica glass in depth, while crystallization process of silica is accompanied. After several slow annealing cycles, the rearrangement of liquid reaches a stable state, which effectively decreases the residual internal stress of the silica fiber. As shown in Figure 8b, compared with the heating and cooling curve in red, the hysteresis of heating and cooling process in black has been significantly decreased. It means the thermal repeatability of FBGs is improved. Therefore, the repeatability of sensor responses to temperature cycles is greatly enhanced by repeated high-temperature annealing operations, and eventually the FBGs are stabilized at temperature up to 1100 °C or even higher.

To date, there have been mounts of developed FBG-based sensors for high temperature measurements. The most concerns on the properties study of FBGs. The Table 2 summarizes the temperature and spectral properties of several types of FBGs mentioned above.

In the field, particularly for well-logging, the FBG sensors need to be placed within the well hole for temperature and pressure measurements. The packaging and adhesive materials, which protect the FBG and attach it to the mechanical structure, are also the key factors determining the detection result, although the FBGs above can withstand the down hole’s temperature lower than 400 °C. Therefore, the further attention should be paid to the development of highly temperature-resistant materials in addition to the FBGs themselves. Our group has produced smart materials for packaging FBG. This will be discussed in the following section.

### 2.3. High Pressure Sensors

Downhole pressure measurement has been in existence since the 1930s, when mechanical gauges were the only available devices [159,160,161]. The readout of these mechanical devices was by a stylus that converted the pressure-induced movement into scratches on a cylinder of soft brass foil. The mechanical gauges were robust and simple but their accuracy was lower than 40 psi. In the 1970s, instead of the mechanical gauges, electronic pressure gauges, based on simple strain-gauges, were developed. These gauges are small, fast and reliable for downhole pressure measurement. However, their resolution and stability were still inadequate, and further improvement was needed. Since then, other pressure gauges have been developed, including sapphire pressure gauges, capacitance gauges, quartz crystal-based gauges and combinable quartz gauge. However, these pressure gauges have drawbacks similar to those of the thermometers mentioned above. These drawbacks provide a good opportunity to employ fiber sensor instead of conventional strain-gauges because of fiber sensors’ unique properties. To date, diverse fiber-optic sensors have been proposed for the downhole pressure measurements, such as DTS-based pressure technique [162], extrinsic and intrinsic Fabry-Perot interferometers [163,164]. FBG sensors possess many benefits over extrinsic fiber devices, as described in the previous section. Actually, a naked FBG is incapable of the downhole pressure measurements because of its low mechanical strength and low intrinsic pressure sensitivity. FBGs need to be packaged securely for mechanical protection and employ transducer structures to improve their sensitivity to the oil or water pressure.

**Figure 9:**
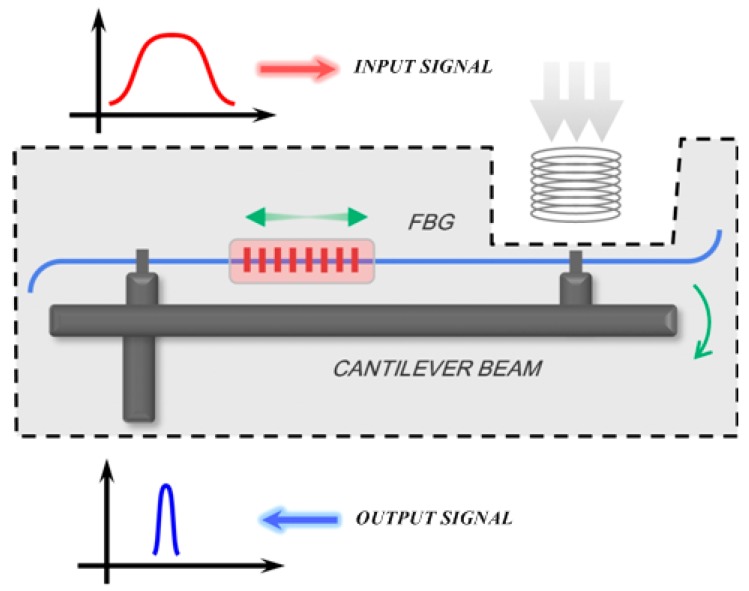
Schematic diagram of FBG pressure sensor.

Previously, we fabricated, the robust and highly sensitive pressure sensor shown in Figure 9. A FBG is attached to the surface of a cantilever beam sealed into a metal box or sheath. A small bellows, which replaces part of the wall of the box, is connected to the free end of the beam. The oil and gas down the well applies pressure on the bellows and consequently deforms the beam. The attached FBG is stretched with the bending of the beam. The method effectively protects the FBG by preventing the oil and gas coming in direct contact with the FBG, as well as improving the FBG’s low intrinsic response to the pressure. Besides, the cast-iron sheath helps the FBG to withstand the high pressure up to 100 Mpa.

**Figure 10:**
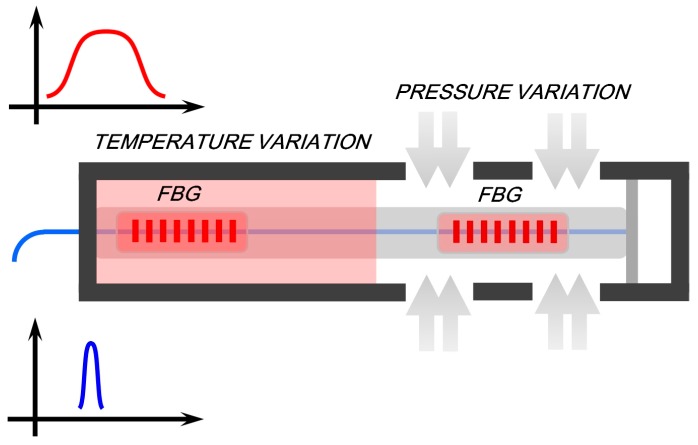
Schematic diagram of FBG sensor for simultaneous temperature and pressure measurements simultaneously.

Pressure is measured in the transmission or reflection spectrum of the FBG by detecting shifts in the peak of the Bragg resonance. However temperature cross-talk can degrade the accuracy of the pressure measurements. One solution is to use a cascaded dual-FBG structure to measure the temperature and pressure simultaneously [165]. An example is shown in Figure 10. The device comprises two cascaded FBGs and three interconnected chambers. A FBG, sealed in the left metal chamber, only measures temperature. The second concatenated FBG is positioned in the open middle chamber, which is open to the surrounding medium. The third chamber is sealed by a membrane, which acts like a piston. The second of the two gratings which is attached to the diaphragm, measures both pressure (as the diaphragm responds to the oil or gas pressure) and to temperature. Under the assumption that the structure does not undergo plastic deformation, these measurements can be linearly related to the parameters applied on each FBG, so that the temperature and pressure can be derived by the vector functions of the wavelengths versus the parameters.

**Figure 11:**
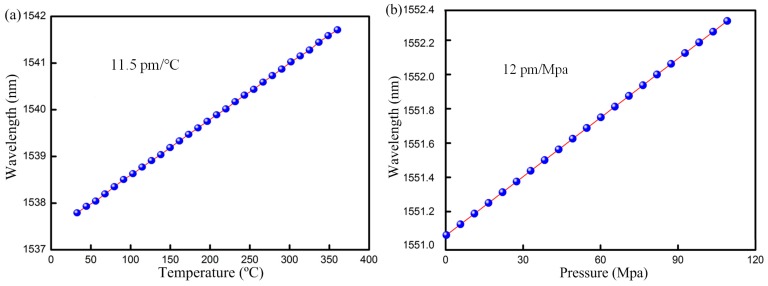
FBG sensor peak wavelength versus (**a**) Temperature; (**b**) Pressure.

Figure 11 shows the highly linear response of this sensor to pressure up to 100 Mpa and temperature up to 350 °C. The results confirm the suitability of such FBG sensors for monitoring downhole temperature and pressure.

The pressure measurement in high-temperature circumstances has always played a critical role in a wide range of industry applications. For instance, the range of operation temperature in energy industries, such as power stations, coal-fired boilers, is usually varied beyond 400 °C, even more than 1000 °C. For these high-temperature circumstances, FBG sensors are able to provide precise and reliable gas pressure detections for safe industrial operations. Since microstructure fibers possess unique geometry and optical properties, air-hole microstructured fiber based FBG sensors have been good candidates for high-temperature pressure measurement [166,167,168,169]. When the external pressure is applied on the fiber hole, these air holes deform accordingly. The induced internal stress birefringence in fiber core contributes to the split of the FBG peak into orthogonal polarizations. When external pressure is increased, the FBG peak split varies with decreased birefringence. Thus, the high-temperature pressure can be detected by monitoring the response of the FBG peak separation. Compared with conventional FBGs, FBG sensors using air-hole microstructured fibers for high-temperature measurements can achieve the discriminated measurements of temperature and pressure, and particularly, perform better for transverse stress. Chen et al. inscribed type II grating in two-hole optical fibers by using an ultrafast femtosecond laser, which is employed to measure high-temperature pressure above 800 °C [166]. Hydrostatic pressure detection from 15 to 2000 psi is obtained with stable and repeatable sensing operation. But the grating linewidth and laser-induced birefringence in the inscription process need to be further optimized for better pressure sensing range and resolution. As another high-temperature resistant application, thermally regenerated FBGs in air-hole microstructure fibers were employed for high-temperature pressure measurements by Chen afterwards [167]. This regenerated FBG from the type I seed grating performs stable at 800 °C and also possesses narrow linewidth and less intrinsic birefringence. External pressure from 15 to 2400 psi at 800 °C is measured by interrogating pressure-induced separation of grating peaks. These approaches based on air-hole microstructure fibers provide a multiplexed single fiber proposal for high-temperature pressure measurement.

**Figure 12:**
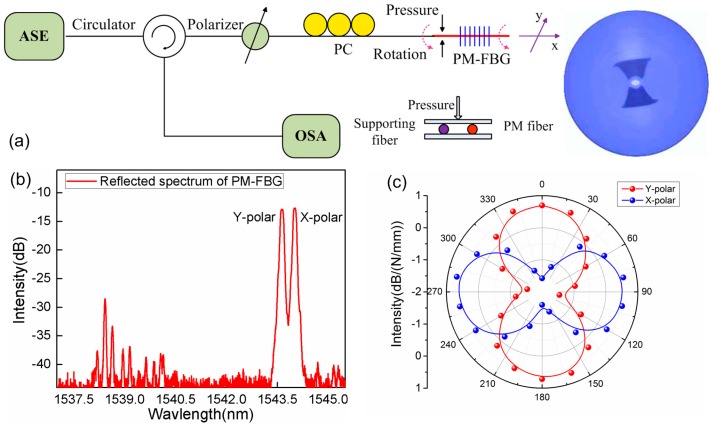
(**a**) Experimental setup for vector pressure measurement; (**b**) Reflection spectrum of PM-FBG with two polarized mode resonances; (**c**) Orthogonal responses of two polarized mode versus transverse stress.

The FBG sensors present the excellent potential for pressure measurement but are not sensitive to the application direction of pressure. Here, we discuss a 2D pressure sensor. The sensing device comprises a section of polarization-maintaining fiber containing a FBG. Two resonances corresponding to two orthogonal modes are reflected by the FBG along the fast- and slow-axes of PM fiber, as shown in Figure 12b. The pressure testing is operated by applying the transverse strain on the FBG. A 25 mm-long uncoated PM fiber located about 10 mm away from a FBG is placed between two glass plates, and another supporting PM fiber is mounted parallel to sensing PM fiber at a distance of 15 mm, as shown in Figure 12a. Local pressure is exerted on the upper glass plate, which is calibrated by a commercial piezometer. The initial E-field intensities of LP01(x) and LP01(y) modes are equal when the LP light is launched into the PM fiber at 45° with respect to one birefringence axis of the PM fiber by regulating the polarization controller. When the transverse strains from 0 N/mm to 1 N/mm are applied on sensing PM fiber, an obvious E-field intensity conversion of LP01(x)-to-LP01(y) mode occurs in the reflection spectrum.

The sensing mechanism can be characterized by the different behaviors of the E-field vector transmitting in the PM fiber under transverse strain. In general, LP light launched into SMF can be decomposed into two orthogonal LP polarization modes, LP01(y) and LP01(x). The E-field orientation of input LP light is maintained throughout the entire SMF, regardless of the transverse strain exerted at a certain direction because of its fully circular-symmetric structure. However, the polarization evolution in the PM fiber is closely related to the perturbation of anisotropy along the fiber axis. When the transverse stress is applied on a short PM fiber section, the resulting anisotropic strain deforms the geometrical cross-section of PM fiber, which changes the refractive index of fused silica due to the photoelastic effect. The refractive index change along the direction parallel to the direction of the force is different from that perpendicular to the direction of force. Consequently, a stress-induced linear birefringence modifies the intrinsic birefringence of PM fiber, which is equivalent to the spatial orientation rotation of the LP E-field. The coupling of orthogonally polarized modes changes with the transverse strain, resulting in the intensities redistribution of polarized modes. Finally, once the PM-FBG is used as pressure sensor, the intensities of the polarized mode resonances present strongly orthogonal responses to the pressures in the different directions. Figure 12c demonstrates the angular dependence of pressure responsivity of the PM-FBG.

### 2.4. Acoustic Wave and Ultrasound Sensors

Acoustic wave detection is another way for achieving seismic information of oil and gas field, which has attracted the attention of the researchers. Currently, the geophones are moved to another site for fresh exploration after one field measurement is completed. Compared to conventional explorations, the distributed permanent monitoring, as a future technical goal in in-situ monitoring or nondestructive testing, can achieve the long-term field monitoring and ensure the consistency of seismic data. However, during the on-line continuous monitoring, these sensors may suffer from various kinds of harsh circumstances and it is difficult to assure the accuracy of field data. Ultrasonic wave (UW) detection is one of the critical methods for nondestructive testing in seismic physical models; this method effectively bridges the theory and field-scale experiments and enables us to obtain the acoustic responses in the absence of a rock matrix in a nearly ideal setting [170,171,172]. The UW detection is traditionally performed by current-driven piezoelectric transducers (PZTs) [173,174]. However, these transducers have some inherent drawbacks: the sensitivity reduces as the diameter decreases; the production materials are generally sensitive to electromagnetic disturbances that result in poor signal-to-noise ratios; and the amplitude-frequency characteristic only offers a narrow detection bandwidth because of strongly resonant effects. In order to provide sufficient contrast between conventional PZTs and fiber-optic sensors, an additional table is given as follows in Table 3. Utilizing the optical means for the UW detection instead of the PZT is good solution. The recovery of the FBG wavelength information is one typical method for characterizing the low-frequency vibration/acoustic. However, to date, it has been impossible to use the wavelength interrogation for retrieving the ultrasonic signal because of the ultra-high acoustic frequency of UW in the imaging of seismic physical model. Spectral side filtering technique has been developed as a method for ultrasonic detection using FBG. The interrogating laser wavelength is set to the linear slope of the FBG’s Bragg reflection resonance and intensity-referenced demodulation yields the acoustic signal [175,176]. The sensitivity of this optical technique is determined by the slope (or spectral bandwidth) of the Bragg resonance. Although the slope can be narrowed by increasing the length of the FBG, the detection sensitivity is significantly reduced when the length of the FBG is larger than the UW wavelength [177]. Fiber-ring lasers using FBGs as filters constitute another method to create a narrow reflection spectrum, based on the strong intracavity mode competition [27,171,178]. For this technology, it is hard to maintain the laser’s power stability, resulting in the large background noise during UW detection. In comparison, a FBG with a π-phase shift (π-FBG) in its center has an extremely sharp notch in the reflection spectrum, making it a good candidate for UW detection [179,180,181].

In contrast with PZT, the response of the FBG-FP UW sensor is essentially non-resonant, but is inevitably influenced by the grating geometry with respect to the UW field. The response sensitivity is significantly determined by the ratio between the length of the grating and the wavelength of the UW owing to the following reasons.

**Figure 13:**
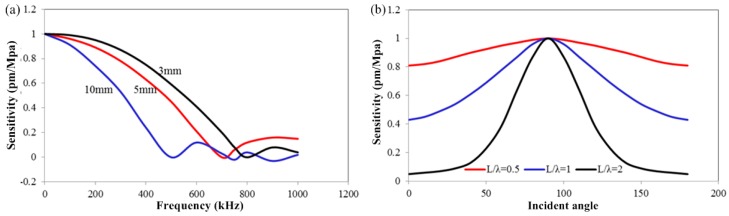
Simulation results: (**a**) FBG length versus response sensitivity; (**b**) Detection direction versus response sensitivity.

The wavelength shift sensitivity *S_λ_* for UW detection can be written as [182]:

(8) $$S_{\lambda }\left(\lambda_{S}/L,\varepsilon_{m}\right)=\frac{\Delta \lambda_{US}\left(\lambda_{S}/L,\varepsilon_{m}\right)}{\lambda_{B0}\varepsilon_{m}}=\frac{\Delta \lambda_{US}\left(v/f_{S}L,\varepsilon_{m}\right)}{\lambda_{B0}\varepsilon_{m}},$$

where Δ*λ_US_* is the shift of the grating, *λ_S_* is the wavelength of the UW, *L* is the length of the FBG, *ε_m_* is the displacement amplitude of the UW, *λ_B_*_0_ is the unperturbed Bragg resonance wavelength, *ν* is the UW velocity in the fiber, and *f_S_* is the UW frequency. The equation clearly shows that the UW sensitivity of sensor is a function of the ratio *λ_S_*/*L*, i.e., it is highly associated with the *λ_S_* or *f_S_*. According to the theoretical analysis in [172,182], in particular, there are three main operating regions as follows: the first region corresponding to *ν*/*f_S_L << 1*, where *S_λ_* approaches zero, so the grating response is practically insensitive to the UW. The second region, corresponding to *ν*/*f_S_L* ≈ 1, where *S_λ_* increases with the ratio *ν*/*f_S_L*, the third region, corresponding to *ν*/*f_S_L >>* 1, where *S_λ_* approaches a maximum value. The simulations in Figure 13 illustrates the discussion above, where the increasing length of the FBG decreases the response frequency band of the FBG. Besides, the detection orientation is another factor to influence the ultrasonic response of the FBG. As shown in Figure 13b, the FBG presents different sensitivities to the UW in different directions. Evidently, the sensor must be fixed in a suitable direction to get high SNR signal.

In the following section, we overview several ultrasonic sensors formed by the different FBGs, as shown in Table 4, and their applications for the imaging of the seismic physical models. Actually, it is important to package the FBGs and then make them sturdy for the scanning operation in the experiments. The packaging methods will also affect the sensitivities, spatial resolution and orientation dependence of the FBG sensors.

As mentioned above, a FBG with a π-phase shift in its center has an extremely sharp notch in the reflection spectrum. With the spectral side band filtering technique, the π-FBG is able to be as smart component for UW detection. In one experiment, in order to improve the fiber’s strain response along the fiber axis, the π-FBG was etched down to 50 µm.

**Figure 14:**
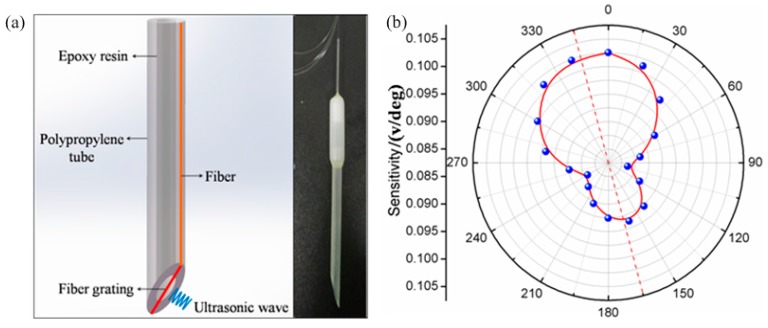
(**a**) Scheme diagram of π-FBG sensor structure; (**b**) π-FBG probe’s responses versus ultrasonic directions (this figure is concluded by recording the peak power of ultrasonic signals in the different detection directions).

In general, there are two methods for realizing the coupling of UW-to-fiber. One is that UW is coupled to fiber through the fiber end-face, so that the efficiency is determined by the materials and size of the fiber end-face. The other is that the UW is applied laterally to the fiber. For the longitudinal wave, the latter method is more effective. In our group’s work, a π-FBG is mounted across the middle of tilted end face of a plastic tube, where the both ends of π-FBG are attached on the side of the tube, as shown in Figure 14a. The UW impinges directly on theπ-FBG (it is not transmitted along the fiber to avoid transmission loss). Because of the asymmetrical structure, the probe presents different sensitivities to the ultrasonic wave impinging from different directions, as shown in Figure 14b. The orientation-dependence can help tolocate the UW source.

**Figure 15:**
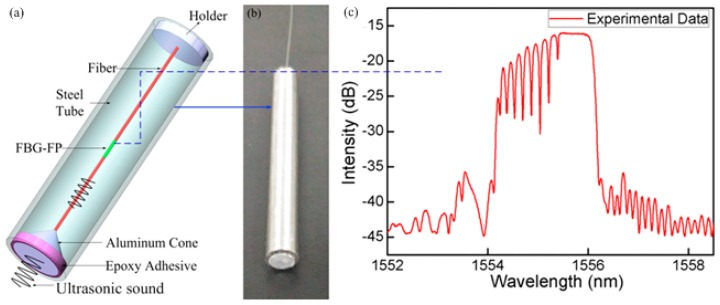
(**a**) Scheme diagram of directional FBG-FP sensor structure; (**b**) Photo image of the sensing probe; (**c**) FBG-FP spectrum with several interference dips overlapping on the top of the FBG resonance spectrum.

In another experiment, we developed a more compact structure employing a 5 mm-long FBG-based Fabry-Perot interferometer (FBG-FP), as shown in Figure 15, to detect the ultrasonic wave instead of the PS-FBG. There are several notches on the top of the reflection spectrum of the FBG-FP, as shown in Figure 15c. High writing laser energy and hydrogen-loading the fiber contributes resulted in large RI modification, contributing to high step-index profiles at the both ends of the inscribed of grating region. The Fabry-Perot interferometer is created by the high average index step at each end of the grating, resulting in Fabry-Perot fringes superimposed on the FBG resonance spectrum. Because their fringe contrasts of interference spectrum are different, it results in a different 3 dB in the bandwidth or the full width half maximum. We selected one FP notch with a 3 dB bandwidth of 8 pm for detecting ultrasonic waves.

In the process of UW detection, it is sometimes not necessary to know the position of the UW source. The FBG can be sealed to provide protection from the hostile environment. As shown in Figure 15a, another sensor structure is formed as the follows. One end of the sensing fiber is mounted into the tip (with a hole) of an aluminum cone. A steel tube with a radius of 3 mm is used to package the sensing fiber. The cone tip is imbedded into the tube and is held by an epoxy resin adhesive. The other end is fixed in a hole through the middle of an aluminum plate embedded into the tube. A certain thickness of epoxy resin adhesive mixed with 5% tungsten powder is coated on the inside surface of the tube and the conical surface to absorb the stray and parasitic ultrasonic waves. The etched FBG-FP is pre-stressed and suspended in the middle of the steel tube. This sensor design presents an orientation-independent performance, which couples the UW energy component in the axial direction of the FBG-FP sensor, while cutting off any unwanted acoustic waves coming from stray reflections from surfaces present in the experimental test setup. During measurement, the directivity of the sensing probe only detects the reflected signal of the models, which is important to achieve a high spatial resolution, especially for the small defects in the model.

**Figure 16:**
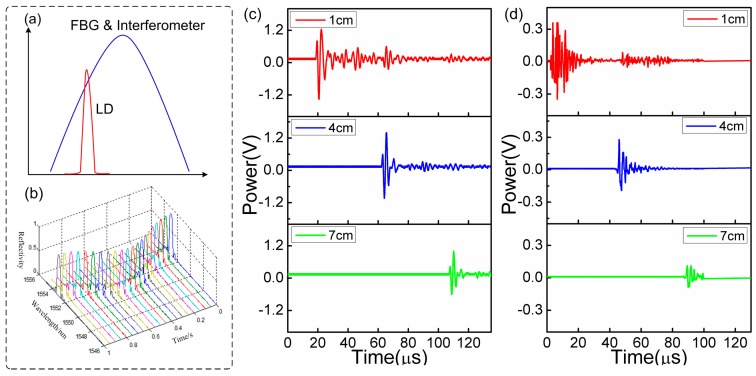
(**a**) Spectral side-band filtering mechanism; (**b**) FBG spectral response to ultrasonic; sensor’s responses to the pulse ultrasonic under the different distances and with different frequencies: (**c**) 300 kHz and (**d**) 1 MHz.

In the experiment, the models detected are placed in water to better index match the UW to the sensor. Since the seismic model is a reduced size of geology structure in equal proportion, the UW with frequency from 100 kHz to 10 MHz is usually employed to the imaging of the models. Here, we choose the sources providing 300 kHz and 1 MHz pulse UWs. In the experiment, the ultrasonic is partially reflected by the interface between air and physical models, e.g., the upper surfaces of models. Meanwhile, the models of Plexiglass materials allow the partials of ultrasonic passing over, and then being reflected by the defects in models, especially by the bottom surface. According to the information of the ultrasonic transmission velocity in water (1500 m/s) and models (2700 m/s), and the time flight in detection result, the thickness and defect positions can be determined. As the UW is applied to the FBG, the resonance spectrum shifts as shown in Figure 16b. The spectral side band technique transforms wavelength information into intensity information that is easier to measure and record. Figure 16c,d demonstrate the time domain spectra changes with the increasing distances at the fixed ultrasonic frequencies of 300 kHz and 1 MHz. The sensor is highly sensitive to the ultrasonic waves at the two frequencies. This result confirms the wide-frequency-band ultrasonic detection capability. With the increasing propagation distances, the detection voltage signal significantly decreases owing to the large loss of the ultrasonic energy in water.

The ultrasonic imaging of the seismic physical models is realized by grating sensor scanning the models and collecting the ultrasonic signals reflected from the models. From the processed we can reconstruct two-dimensional images of the models, thereby showcasing ultrasonic imaging capabilities of the FBG sensor.

**Figure 17:**
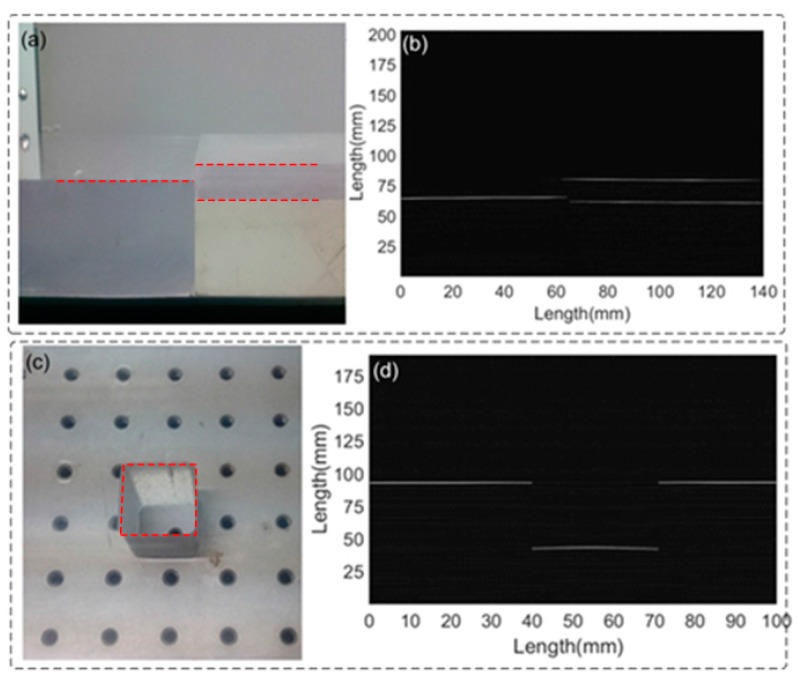
(**a**) Photograph of the step-type Plexiglas model; (**b**) UW image of the physical model; (**c**) Photograph of the sunken plexiglas model; (**d**) UW image of the physical model.

Figure 17a shows a step-type Plexiglas block that includes three interfaces. The thickness of the upper model is approximately 2 cm. Figure 17b demonstrates the UW imaging of the model, as expected, which clearly presents the surfaces of the model. The result is also in agreement with the shape and structure of the actual model. It is noted that the image of the upper model is longer than that of the actual one. This fake extension mainly contributes to the UW diffraction of the interface and the highly spatial resolution of the FBG-FP probe.

The second model is a sunken with a length and a width of 3 cm and 2.5 cm, respectively, in a larger rectangular Plexiglas block model, as shown in Figure 17c. The repeated scanning process is used for the 2D imaging of the model. As shown in Figure 17d, the rectangular hole is clearly performed as expected, in which the edges are clearly separated and reconstructed. It is similar to the image of the step-type model above in that the weak diffraction wave also appears. This effect makes the image of the bottom slight larger than that of the actual model.

## 3. Downhole Applications

**Figure 18:**
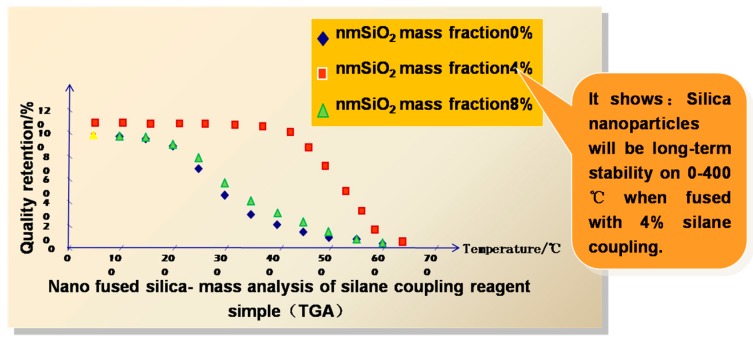
Testing result of the glue, the sample with the particles of 4% nanometer SiO_2_ is the optimized one.

In the above section, the temperature and strain measurement properties of FBGs have been discussed. For oil and gas fields, the naked FBGs are unable to measure the physical parameters in the harsh environments within wells. The gratings must be supported and protected by sturdy packaging techniques, with attention to the materials and sensing structures. The long-term stability and repeatability of sensors in the downhole applications are determined by the packaging techniques, with the exception of the performances of the FBGs themselves, as discussed above. Our group employs the alloy material of Nb-40 Ti-5.5 Al for FBG packaging. The benefits of the material are the small elastic modulus, erosion resistance to H^+^, Cl^−^, CO_2_, H_2_S under well, and especially the high temperature and high pressure resistances. The packaging structure of the sensor is fabricated using the alloy, after which, the FBG is fixed in the metal structure using customized specific glue of a silane coupling agent into which 4% by volume of fused SiO_2_ nanoparticles are mixed. The mixing ratio is controlled to optimize the properties of the glue. Compared with the polypropylene rubber adhesive, the custom glue can maintain long-term good stability over the temperature range of −20 °C to 400 °C. The corresponding testing results have been finished in the experiment, as shown in Figure 18.

**Figure 19:**
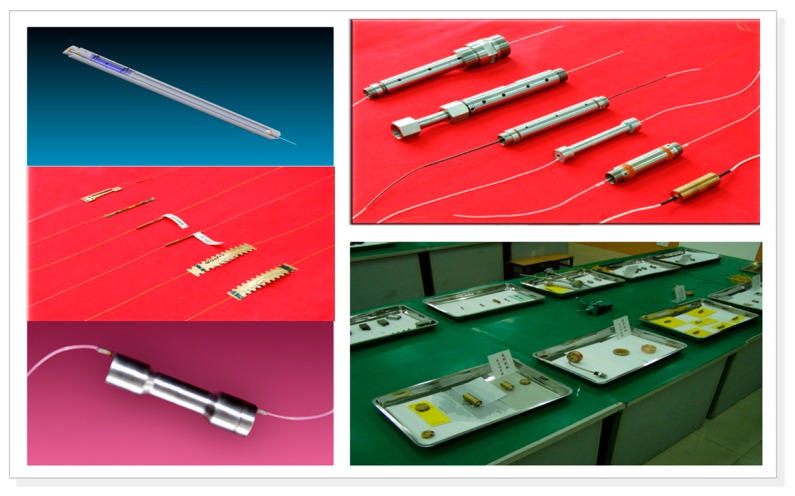
Series of FBG sensors, including strain sensors, high temperature sensors, high pressure sensors, and accelerometers.

Our group has fabricated series of FBG sensors using in house materials and components, as shown in Figure 19. Among the sensors, there are some post-process operations that are employed to improve the temperature sensitivity and pressure sensitivity of the FBGs as well as prevent detrimental the cross-talk between measurement parameters. In the well-logging, these sensors are utilized to measure the gas, oil and water pressure and temperature within wells, so they must maintain tight seals to protect the delicate fiber gratings.

**Figure 20:**
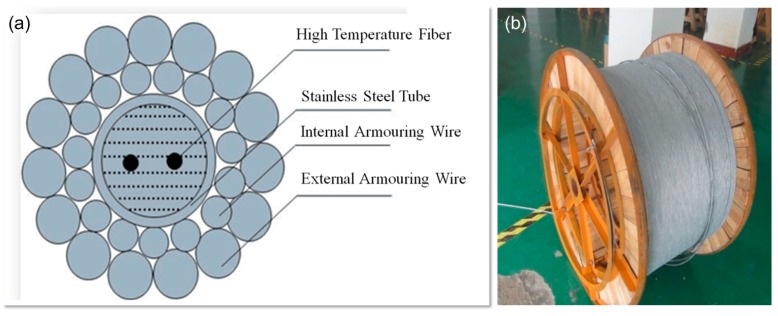
(**a**) Cross-section structure of downhole cable; (**b**) Photo of the 10 km-long downhole cable.

For sensing and logging vibration, temperature, pressure and acoustic waves down deep wells, it is necessary to use sturdy fiber cables that can also withstand the high temperatures and pressures and support their own weight. Our group has made a strong downhole cable using the special design and materials. The fiber in the cable is coated with metal and polyimide that can withstand the high temperature. The cross-sectional structure of the cable is shown in Figure 20a. Now we are collaborating with a commercial cable manufacturer to produce 10 km-long cables, as shown in the photo in Figure 20b.

**Figure 21:**
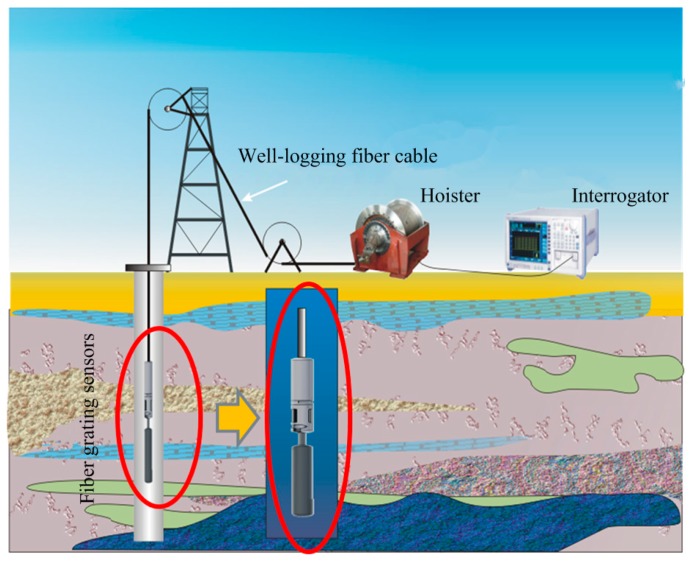
Schematic diagram of the downhole testing.

In September 2010, our team of 16 members went to Liaohe Oilfield of Qi-40-Guan-23 well for the downhole testing, which aimed to measure the performance of downwell optical cable, fiber grating sensors, and connectors under downhole harsh environment, such as high temperature, high pressure, and strong corrosion (As shown in Figure 21). A series of high-temperature and high-pressure fiber grating sensors based on the high-pressure FBG sensor in Figure 10 of Section 2.3 were fabricated, which possessed obvious advantages, such as compact size, low cost, wide detection range, high sensitivity (temperature: 0.1 °C, pressure: 0.05 MPa), corrosion resistance, high security. These sensors can detect the temperature (measurement range: 0~350 °C) and pressure (measurement range: 0~100 MPa), simultaneously. These home-made fiber grating sensors were also employed to detect the varying ranges of downwell temperature and pressure.

**Figure 22:**
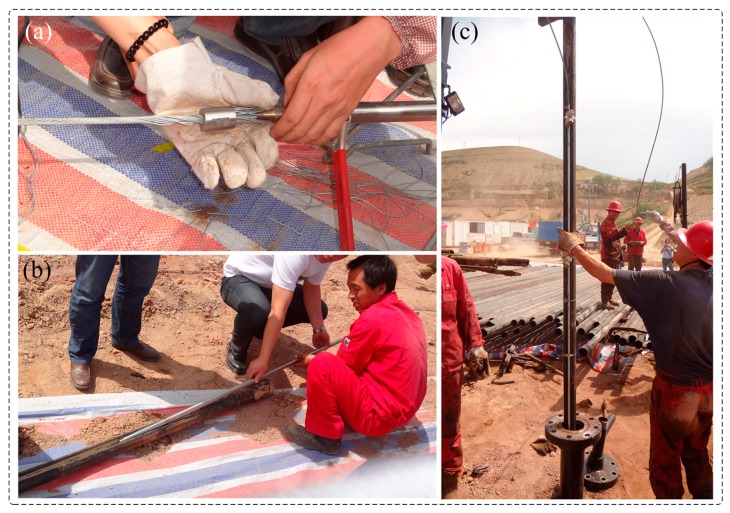
Photos of well-logging field: (**a**) Downhole cable; (**b**) Tied to the downhole tube; (**c**) Sensor down well process.

Before downhole logging, the necessary preparation works is completed. The sensor is connected with the home-designed cable that is also the key factor to determine the results. The armored cable contains a dual-layer tube to make sure the safety of fiber. The aramid fiber coats surround the tube, named buffer layer, which has the function of slowing down the external shock. The galvanized steel wires forms two-layer shell to further protect the cable. As the FBG sensor is connected with the cable, they are tied to the downhole tube using metal hoops. When everything is ready, the sensor is lowed into the well hole (shown in Figure 22), and the monitoring instruments on the surface monitor the temperature and pressure variations.

**Figure 23:**
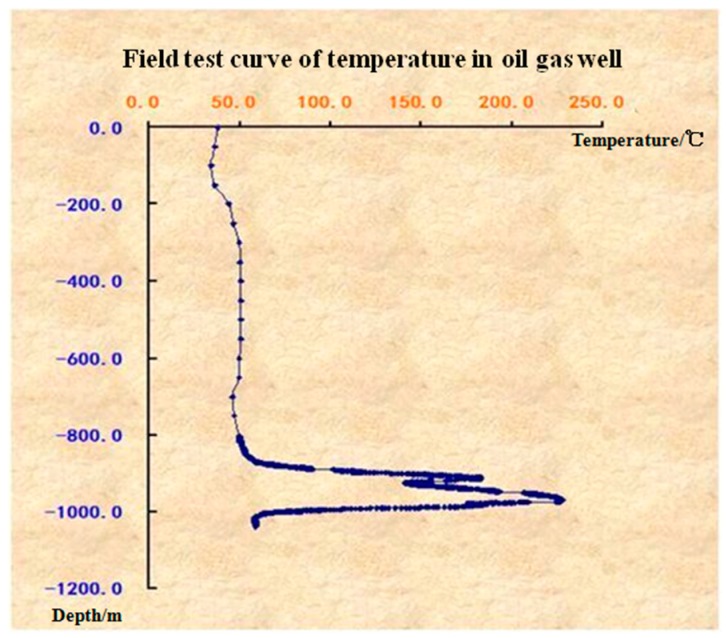
Temperature distribution down to 1100 m depth of well hole, measured by FBG sensors.

The following Figure 23 shows one of the testing results, the recovery of the temperature profile of the well from the ground level to the 1100 m below the surface. It is clearly seen that, in the downhole region from 800 m to 1000 m, there is large temperature spike, to a maximum of 240 °C which is very much higher than the approximately 40 °C to 50 °C between the surface and 800 m depth. This result is agrees well with that temperature profiles measured in this oil field using other thermometers, providing confirmation that our group’s FBGs, sensors, packaging materials and cables are capable of oil well logging.

**Figure 24:**
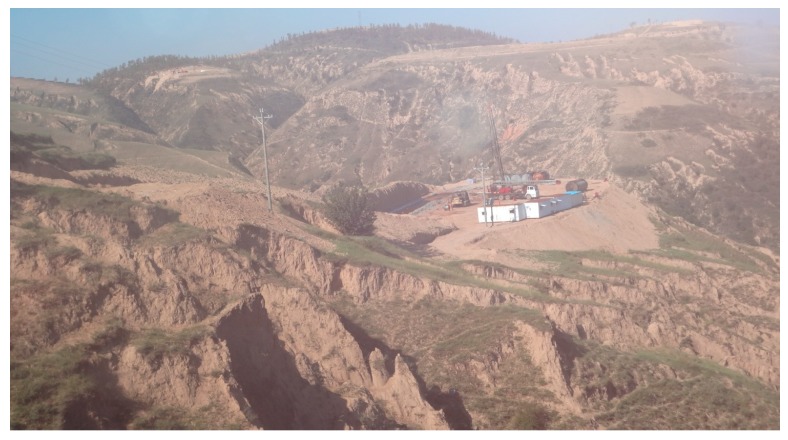
Photo of Oilfield test in Jinbian.

On 6–15 July 2012, our group, together with China Petrol Logging (CPL) Drilling Center and Institute of the Changqing Oilfield tested our FBG sensors for temperature and liquid level measurements in an oil field. The well, situated in Jinbian County, Yulin City in Shaanxi Province, China, required testing to a depth of about 1400 m, as shown in Figure 24. On the basis of the previous downhole testingin Liaohe Oilfield, these home-made fiber-optic grating sensors, with improved temperature and pressure sensitivities of 12.9 pm/°C and 30.9 pm/MPa, respectively, were employed to implement the discriminated measurement of temperature and pressure for the oilfield test in Jinbian. Both the packaged fiber liquid level sensors and downhole cables can withstand the operating temperature of 150 °C and pressure of 40 MPa. The resolution parameters of the demodulating system were 0.1 °C (temperature) and 5 m (liquid level). A monitoring system was employed to provide the information of alarm, machine halt, and reset according to the downhole depth.

**Figure 25:**
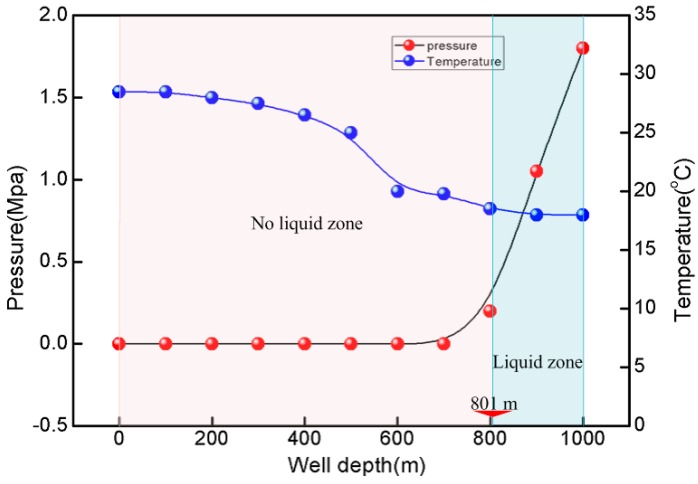
Downwell temperature, pressure and liquid level distribution.

Figure 25 shows the downhole temperature, pressure and liquid level information from the ground to the 1000 m depth. As can be seen, the temperature varies from 18.4 °C to 29.8 °C and the pressure changes from 0 Mpa to 1.87 Mpa in the downhole region from ground to 1000 m, which are also well agreed with that measurement using other thermometers and pressure gages. The liquid level zone is undetected until the downhole depth over 801 m, which is also marked in Figure 25.

The detailed measurement information of the latest field tests by our FBG sensors is listed as follows in Table 5.

## 4. Conclusions

### 4.1. Research Foundation and Statues of Our Group

The main research activities of our group concern nonlinear optics, photo electronic technology, optical fiber sensing technology and their applications in the fields of well-logging, oil and gas pipeline monitoring, and downhole seismic exploitation, as the follows.

- In-fiber grating inscriptions into diverse fibers using the femtosecond infrared and UV laser light.
- Micromaching fibers using femtosecond laser and operation high precision mechanical stages.
- Physical, biomedical and chemical sensors using FBGs and interferometers.
- Full fiber light sources for sensing system.

### 4.2. Problems and Future Directions

FBG sensors have been under development for several decades. FBGs are an enabling technology for oil and gas logging and health monitoring of pipeline applications. FBGs offer the possibility of sensitive, non-destructive, long-term in-situ measurements of temperature, stress, strain, deformation and vibration in harsh environments. Novel applications for on-line monitoring and service lifetime in a variety of applications and oil and gas fields are made possible by FBGs. Although the FBG sensors have attracted the great interests of the oil and gas industry and presented amazing application prospects, there are still some techniques that are need further research and development:

- Novel sensing fiber and FBGs, providing high sensing performances
- Mechanics of host material/fiber interfaces
- Adequate fiber coatings and packaging designs
- Sensor design and configuration
- Long distance downhole cable

### 4.3. Fiber-Optic Sensor Standardization

In the oil and gas industry, diverse fiber-optics sensors have appeared and been applied in the fields of the oil and gas well-logging, seismic exploration above ground and in deep wells, and health monitoring of pipelines. It may be a good time to promote the industrialization of fiber-optic sensors in the petroleum industry, however without a degree of standardization, there is likely to be considerable confusion. An extensive search of the literature shows a great diversity of methods, definitions and approaches. There is an urgent need for establishment of fiber optic sensor standards. Bearing this in mind, researchers should promote the properties of the fiber sensors by solving some key scientific and technological problems, to give fiber sensors the best possibility of widespread applications in the oil and gas industry. We believe that fiber-optics sensors standardization is key to the successful promotion of smart optical technology in industry.

## Figures and Tables

**Table 1:** FBG basedaccelerometers.

Type	Structure	Orientation Dependence	Sensing Spectrum Bandwidth	Sensitivity	Frequency Range
Mechanical Transducer [117,118,119]	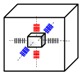	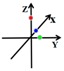	>0.6 nm	0.123 G	5 Hz~2.5 kHz
TFBG [110,120]	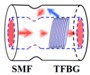	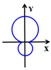	>10 nm	0.25 G	5~250 Hz
PM-FBG [112,113]	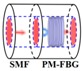	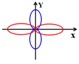	>5 nm	0.12 G	10~30 Hz
DBR [121]	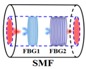	Very small	0.54 nm	1.87 × 10^−3^ G	14.6 Hz~0.99 kHz
Cladding FBG [25]	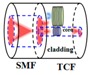	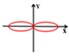	<1 nm	2.778 × 10^−4^ G	6~10 Hz

**Table 2:** FBG-based high temperature sensors [138,139,140,141,142,143,144,145,146,147,148,149,150,151,152,153,154,155,156,157,158].

Type	Sensitivity	Range	Reflectivity	Bandwidth (3-dB)
FBG (UV)	7.53–13 pm/°C	0–600 °C	20%–100%	<1 nm
RFBG	11–17.7 pm/°C	20–1100 °C	9%–40%	0.1–0.2 nm
FBG (Femtosecond laser)	11.5–25 pm/°C	20–1100 °C	80%–98.5%	0.1–7 nm

**Table 3:** Comparison between PZT and fiber-optic ultrasonic sensor.

PZT	Fiber-Optic Sensor
Narrow detection bandwidth	Broadband response
Large size (diameter or length in millimeter range)	Small size, light weight, flexibility
Sensitive to electromagnetic disturbances	Immunity to electromagnetic interference
Inadequate resistance to high temperature (Curie temperature, *T_c_* < 60 °C)	High-temperature resistance (operating temperature > 600 °C)
Poor stability of signal transmission (~10 m)	Stabilized transmission in fiber (>20 km)
Poor multiplexing	Good multiplexing, multichannel detection
No direction recognition	Vectorial sensing

**Table 4:** FBG-based ultrasound sensors.

Ultrasonic	Coupling Method	SNR	Frequency Bandwidth
PS-FBG	Fiber end-face	19.55 dB	20 kHz~10 MHz
FBG-FP	Etched FBG-FP	27.96 dB	20 kHz~7.5 MHz
FBG	Cone end-face	32.57 dB	20 kHz~2 MHz

**Table 5:** Oilfield test details.

Oilfield Site	Test Time	Continuous Measuring Hours	Well Depth	Pressure Range	Temperature Range
Liaohe Oilfield	15 August 2010	6.5 h	2000 m	0~15 Mpa	36 °C–241 °C
Jinbiancounty, Yulin city, ShaanxiProvince	6–15 July 2012	6 h	1638 m	0~1.87 Mpa	18.4 °C–29.8 °C
